# Supplementary material for: Developing an applied model for making decisions towards the end of life about care for someone with dementia
Source: PLoS One. 2021 May 27;16(5):e0252464. doi: 10.1371/journal.pone.0252464 (PMC8158904; doi:10.1371/journal.pone.0252464)
Supplement: Attachment — Submitted filename: R2 response to reviewer.docx [file pone.0252464.s002.docx]

Dear Dr Frey and reviewers,

We would like to thank the editor and both reviewers for reviewing and checking our revision.

We have provided detailed response to reviewer 1 comment below.

We look forward to hearing from you.

Yours sincerely,

Dr Nathan Davies (on behalf of all authors)

Reviewer 1

The authors state that "We have cited systematic review of decision aids, which covers a range of different decision aids published." However, at least one of these aids, published by Arcand, was not included. What were the creteria for selection of published decision aids and how many more were not included?

# This was not included in the published systematic review as it is an information resource or booklet on comfort care, it is not described as a decision aid in any of the papers that were identified as part of that search, and does not contain the features of a decision aid. This was an exclusion criteria in the published systematic review. However, we agree it is a very important resource to support decisions which has been used in multiple studies and evaluated, we have added the Arcand paper to the introduction of the current paper see line 97.
